# Impact of Transcatheter Aortic Valve Implantation on Kidney Function: the “Renovalvular” Interaction in Aortic Stenosis

**DOI:** 10.36660/abc.20190753

**Published:** 2019-12

**Authors:** Antonio de Santis

**Affiliations:** Universidade de São Paulo Instituto do Coração - Unidade Clinica de Valvopatia, São Paulo, SP - Brazil

**Keywords:** Heart Failure/complications, Renal Insufficiency/ complications, Aortic Valve Stenosis/complications, Transcatheter Aortic Valve Replacement/trends, Risk Assessment.

The advent of renal congestion in heart failure was first described by Frédéric Justin Collet (1870-1966), a French pathologist who found the notion of passive renal congestion related to heart dysfunction, creating the revealing term “*rein cardiaque*” in the early 1900s.^1^ The term cardiorenal syndrome emerged from a 2004 National Heart, Lung, and Blood Institute Working GrouP conference evaluating the complex interactions between the heart and kidney.^2^ The main pathophysiological mechanisms related to this condition are increased central venous and intra-abdominal pressures; reduced cardiac output and cardiac index; neurohormonal dysregulation; oxidative stress and inflammatory mediators.^3^ Degenerative aortic stenosis (AS) represents one of the most prevalent valvular heart diseases and an important cause of heart failure, with a strong correlation with aging process. The combination of atherosclerosis, biomineralization and oxidative stress leads to calcium deposition within the valve leaflets.^4^ The *“renovalvular”* interaction in AS may represent a two-way path, from a pathophysiological perspective. In one way, AS may impair kidney function by arterial hypoperfusion and systemic venous congestion. On the other way, chronic kidney disease (CKD) is also an important risk factor for AS, due to the massive and aggressive calcification of the leaflets, mainly imposed by imbalances in the calcium and phosphorus homeostasis.^5^

For patients with AS undergoing conventional surgical aortic valve replacement (AVR), there is an increase in complication rates such as major bleeding and reoperation when comparing patients with moderately reduced kidney function (estimated glomerular filtration rate [eGFR] between 30-60 ml/min/1.73 m^2^) versus those without kidney disease.^6^ Mortality after surgical AVR also increases with worsening GFR.^7^

The development of the transcatheter aortic valve replacement (TAVR) for the treatment of AS has brought hope for a grouP of patients without effective therapeutic perspective due to their clinical profile, characterized by the presence of clinical frailty and multiple comorbidities, making surgical AVR not feasible.^8^ In this scenario, TAVR represents a potentially less invasive therapeutic alternative for patients with AS and CKD. Some previous studies evaluated the clinical impact of TAVR on patients with CKD. In the classical PARTNER trial, there was a 34.4% 1-year mortality for patients with severe CKD.^9^ For patients undergoing dialysis treatment and TAVR, there was also a higher mortality rate and major bleeding.^10^ Moreover, the occurrence of acute kidney injury in the peri-procedural TAVR period is also associated with poor outcomes.^11^ On the other hand, some previous studies demonstrate a positive impact of TAVR on renal function, especially in patients with moderate to severe CKD, with a significant recovery of eGFR possible related to the improvement of cardiac output and reduction of systemic venous congestion.^12-14^

Promisingly, the present study conducted by Calça et al.,^15^ provides additional data on the positive impact of TAVR on the kidney function. Through a retrospective and unicentric study, 233 patients with AS who underwent TAVR were stratified into 3 groups according to basal eGFR (ml/min/1.73 m^2^): grouP 1 (eGFR > 60), grouP 2 (30 ≤ eGFR < 60) and grouP 3 (eGFR < 30). The renal function was re-accessed one month and one year after TAVR. The authors observed a significant improvement in eGFR in patients with moderate (grouP 2) to severe (grouP 3) CKD (around 15.6% in one year; around 58.6% in one year, respectively). Conversely, patients from grouP 1 had a progressive decline in eGFR one year after the TAVR procedure (P < 0.001 vs. pre-TAVR). Nevertheless, there was a low incidence of dialysis therapy in one year (2.4%). Possible reasons implicated by the authors in this worsening of eGFR in grouP 1 were higher use of iodinated contrast in this grouP (65% of patients) and the use of angiotensin receptor blockers and angiotensin-converting enzyme inhibitors in the post-procedure period. Multivariate logistic regression analysis identified that the use of iodinated contrast was an independent predictor of worsening kidney function, unrelated to the volume of contrast used.

Despite its inherent limitations (single center, retrospective and observational study) the study by Calça et al.^15^ brings a promising light on the impact of TAVR on kidney function in patients with moderate to severe CKD. This pre-procedure kidney dysfunction may not be an exclusion issue for the TAVR, given to the possibility of short- and medium-term improvement. Future randomized, multicenter studies, with a stricter contrast type control and longer, follow uP , are required for a definitive conclusion.
